# Beyond the joystick: deep learning games for hand movement recovery

**DOI:** 10.3389/fresc.2025.1653302

**Published:** 2025-11-12

**Authors:** Vrinda Acharya, Hirakjyoti Roy, Surekha Kamath, Aneesha Acharya K

**Affiliations:** 1 Manipal School of Commerce and Economics, Manipal Academy of Higher Education, Manipal, India; 2 Department of Instrumentation and Control Engineering, Manipal Institute of Technology, Manipal Academy of Higher Education (MAHE), Manipal, India

**Keywords:** deep learning, gesture recognition, hand rehabilitation, health, cognitive function, convolution neural networks

## Abstract

**Introduction:**

This research explores the use of Deep Learning (DL) techniques for hand and gesture recognition to support hand rehabilitation programs. The primary objective is to enhance cognitive function and hand-eye coordination through gamified therapeutic exercises that track and respond to hand gestures in real time.

**Methods:**

Pre-trained Convolutional Neural Network (CNN) models were employed for hand recognition using Google's open-source MediaPipe Library. Four classic arcade games-Pong, Tetris, Fruit Ninja, and a Virtual Keyboard-were redeveloped as gesture-controlled rehabilitation tools within a web-based interface built using the Phaser.js framework. A score-based system was implemented to track user performance and progress. Usability was evaluated using the System Usability Scale (SUS), and statistical validation was performed with a one-sample *t*-test against the industry benchmark.

**Results:**

Data collected from 15 participants demonstrated consistent gesture recognition accuracy and stable game control performance. The SUS evaluation indicated favorable user responses, with usability scores exceeding the benchmark threshold, suggesting that participants found the system intuitive and engaging.

**Discussion:**

The study confirms the feasibility of using monocular camera-based computer vision for hand rehabilitation. Compared to existing low-cost rehabilitation tools, the proposed system provides an accessible, interactive, and affordable alternative. Integrating gesture recognition with gamified interfaces effectively supports dexterity recovery and motivates users through engaging gameplay. These findings establish a foundation for future development of AI-based rehabilitation platforms using standard camera devices.

## 1 Introduction

The human hand can have extensive movements and functions crucial for daily activities. Hand dysfunction can arise from various conditions, including various neurological disorders, hand injury, or due to ageing. The primary goals of hand rehabilitation are to restore movement, strength, and dexterity while enhancing the patient's ability to perform daily activities. Effective rehabilitation programs are designed to meet the unique needs of each individual, ensuring that recovery is both targeted and comprehensive. One of the primary goals of these programs is to improve the range of motion, which is achieved through stretching and mobility exercises that enhance joint flexibility and reduce stiffness. These exercises are essential for restoring natural movement patterns, especially after injury or surgery. In addition to mobility, rehabilitation often focuses on strengthening muscles through resistance training. This approach helps rebuild muscle strength and endurance, enabling patients to perform daily activities with greater ease and confidence. Furthermore, a crucial component of rehabilitation is the enhancement of coordination and fine motor skills, commonly referred to as dexterity. Through tasks and exercises that require precision, timing, and hand-eye coordination, such as picking up small objects, tracing shapes, or gesture-based interactions, patients can regain control over fine motor functions essential for independence in everyday tasks.

Evaluating hand function and the effectiveness of rehabilitation interventions involves various methods. Traditional methods include manual grip strength, range of motion, and functional ability tests, often conducted by occupational therapists. Traditional therapy often faces limitations such as low patient motivation and difficulty in home-based deployment. Motion capture systems and electromyography (EMG) are used to analyze hand movements and muscle activity, offering quantitative data on function and recovery. The advancement of Smart devices such as sensors, wearable sensors, Microcontrollers, and the Internet of Things (IoT) has revolutionized the collection of qualitative and objective information about patients' motor functions at a low cost, enhancing treatment efficiency (1). These platforms use common objects equipped with sensors and displays to monitor patients' activity during rehabilitation exercises based on Activities of Daily Living (ADL), such as drinking, cooking, and cleaning. Moreover, these solutions provide consistent tools for transparent monitoring of ADLs at home, facilitating better management of hand dysfunction. In recent years, game-based, AI-driven interaction in rehabilitation exercises has shown the ability to enhance patient engagement and adherence, with studies indicating they may be more effective than conventional rehabilitation methods (2, 3).

This paper aims to create a simple, easy-to-use web-based application for improving dexterity and hand-eye coordination for ADL tasks. We have reimplemented old arcade games in Python and integrated the controls via hand tracking, enabling a gamified cognitive and dexterity training experience. Simple diagnostic tools and a gamified experience provide an informative and engaging experience. The aim is to use existing deep learning methods to create camera-based applications instead of fantasy equipment, which is often impossible with financial constraints. This also enables low-cost hand-therapy solutions that do not rely on expensive hardware. A web-based interface will enable anyone with an internet connection to use the application, eliminating several constraints. The present work focuses on demonstrating the technical feasibility and usability of a camera-based gesture recognition system for rehabilitation.

## 2 Related works

Krizhevsky et al. (4) introduced Convolutional Neural Networks as a viable and scalable alternative for image classification and object recognition tasks instead of hard-coded and hand-designed features. He et al. (5) modified the CNN architecture proposed in (4) and its successor architecture by introducing the notion of a skip connection. The skip connection (a residual connection) connects convolutional blocks further down the neural net chain and its feed-forward chain. Howard et al. (6) introduced the notion of depth-wise separable convolution, which leads them to greatly improved efficiency gains, thus allowing them to deploy CNNs on smaller devices with constrained hardware resources like smartphones. Sandler et al. (2) improved the existing MobileNet architecture and introduced MobileNet v2. It presents the inverted residual structure, where the skip connections are between the bottleneck layers. This leads to more efficiency gains. The CNN models used in this research are slight variations of this architecture.

Dosovitskiy et al. (3) introduced the Vision Transformer (ViT) architecture, marking the first adaptation of the transformer model for image recognition tasks. However, Zhu et al. (7) demonstrated through detailed experimental analysis that ViTs tend to underperform in low-data scenarios, where Convolutional Neural Networks (CNNs) remain more effective—an insight that supports the use of CNN-based architectures in this research. In parallel, Bazarevsky et al. (8) proposed an optimized version of the MobileNetV2 architecture, which serves as the foundation for the object recognition models employed in this project via the MediaPipe library. MediaPipe Solutions, developed by Google, provides a comprehensive suite of AI and ML tools, including pre-trained models for efficient hand tracking and gesture recognition (9, 10).

Alfieri et al. (11) reviewed recent literature on gamification in musculoskeletal rehabilitation, highlighting its effectiveness compared to conventional therapies. Oña et al. (12) presented a novel system that enhances the assessment and rehabilitation of eye-hand coordination. The system suggested by the authors uses serious games in virtual environments to evaluate hand-eye coordination. Alarcón-Aldana et al. (1) presented a novel system that combines serious games with robotic devices to enhance the assessment and rehabilitation of eye-hand coordination. Panaite et al. (13) concluded that the game-based rehabilitation system has been found to motivate patients to continue their rehabilitation, as they feel rewarded when they win. Wang et al. (14) developed a system that combines AR technology, an air pressure-detecting device, and game-based learning to make the rehabilitation process more engaging and less tedious for patients.

Jha et al. (15) investigated the efficacy of a novel glove-based virtual rehabilitation system named Rehab Relive Glove, designed for patients with post-traumatic hand injuries. The authors aim to address the limitations of conventional physiotherapy and existing virtual rehabilitation technologies by providing a portable, cost-effective, and ergonomic solution that can be used in clinical and home settings. Bouatrous et al. (16) presented a hand rehabilitation game designed with clinicians for hand rehabilitation after a stroke. The game simulates fruit harvesting using a Leap Motion controller for hand tracking and the Unity 3D platform. Bostanci et al. (17) investigated the effectiveness of virtual reality (VR) training in improving manual skills and grip strength in the non-dominant hand of healthy individuals. The study concludes that VR games can effectively enhance hand function and grip strength in the non-dominant hand, suggesting that such interventions could serve as engaging and beneficial tools in rehabilitation settings.

M. Alimanova et al. (18) focused on gamifying hand rehabilitation using Leap Motion technology to create a game that helps train hand muscles through virtual reality tasks mimicking daily life actions. Chiu et al. (19) investigated the effectiveness of the Wii Sports Resort training in improving coordination, strength, and hand function in children with hemiplegic cerebral palsy. Lansberg et al. (20) investigated the feasibility and effectiveness of using the Neofect Smart Glove, a virtual reality device, for unsupervised home-based hand rehabilitation in chronic stroke patients. Luna-Oliva et al. (21) investigated the potential of using the Xbox 360 Kinect as a therapeutic tool for children with cerebral palsy (CP) in a school environment. The study found significant improvements in the children's balance and activities of daily living (ADL) after the video game treatment.

Considering the above literature, we have identified the gap in understanding the utilization of deep learning models for accurate gesture recognition that ensures the system is adaptive, engaging, and capable of real-time processing. This approach can provide an effective and motivating rehabilitation tool for patients with hand function disorders.

## 3 Materials and methods

This research uses the Mediapipe Library (9, 10), particularly the javascript version of the library, for hand tracking and gesture recognition. Deep Learning methods only work when provided with gigantic amounts of data. E.g., the well-known ImageNet dataset consisted of more than 1 million images, and MS-COCO, another well-known dataset for object detection dataset, consisted of more than 320 K images. These datasets were state of the art about 5–7 years ago. The field has since moved on to larger and more diverse datasets, which is a major reason for the success of Deep Learning Methods. Therefore, with these resource constraints, the best approach seems to be not to collect a custom dataset and annotations but to use well-tested open-source packages trained on a huge dataset. The added advantage is that this mitigates any chances of dataset bias for our particular use case.

**Figure 1:**
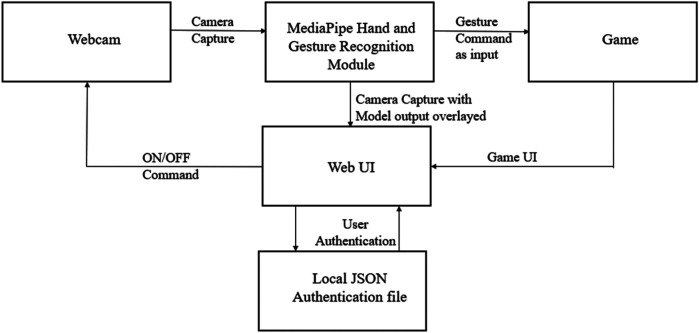
The flow process of the development of the virtual hand-tracking module.

For the hand-tracking task, the camera input is read through the OpenCV javascript library and fed as input to the hand-tracking module from the MediaPipe library, which gives us the required output. OpenCV helps in frame capture and preprocessing. For Gesture Recognition Module Applies a CNN classifier trained to detect gestures such as swipe, pinch, tap, and finger point. PyGame is a desktop game prototyping. This output is then overlaid on top of the input image and displayed to the user. Due to low inference time, this process is nearly real-time. The gesture recognition module used for this research is not the default module provided with the MediaPipe library, but a composite of the hand-tracking module and some custom code. This is because custom gestures are implemented for targeted dexterity exercises. The following block diagram, shown in Figure 1, aims to provide some clarity.

The work consists of different modules, which all talk to each other via the web UI. The host server accomplishes this work. Upon visiting the website, the users are prompted by a login page. The Username and Password are stored in a local JSON file, which is queried by the website server at the time of login. The login process creates a separate profile for each user, enabling a personalized experience as stated in the research objectives.

After successful authentication, the user is directed to a page with a dashboard, which provides the user with an interface to the various modules developed, such as hand tracking, gesture recognition, and playing the games developed. After selecting a particular task, the web UI turns on the webcam. The webcam outputs an image, which is fed into the hand-tracking module. In the case of the hand-tracking module, this output is then overlaid on top of the original image captured by the webcam and displayed on the webpage. If the games are selected, the hand-tracking outputs of the hand-tracking module are taken as inputs to the gesture recognition module, which is automatically turned on, and the gestures recognized are fed as input to the games, which provides a personalized gamified experience to the user. The specifics of the gesture recognition module are discussed in the following chapter.

The user's authentication details are stored in a local JSON file to simplify the authentication process. It must be noted here that this is not a safe process, but it exists only as proof of concept of how each user's personal details may be stored for personal experience. In standard professional settings, this may be done by storing details on a different server, and details encrypted end-to-end; however, this is out of the scope of the effort. Fifteen participants completed the 10-items, 5 point Likert scale System Usability Scale (SUS), after interacting with the hand-tracking games (Pong, Tetris, Fruit Ninja, Virtual Keyboard). The SUS, developed by John Brooke (22), was used to evaluate usability of the game beyond gesture recognition accuracy. The mean SUS score was compared to the benchmark value of 68 using a one-sample t-test to determine significance.

## 4 Games development and discussion

**Figure 2:**
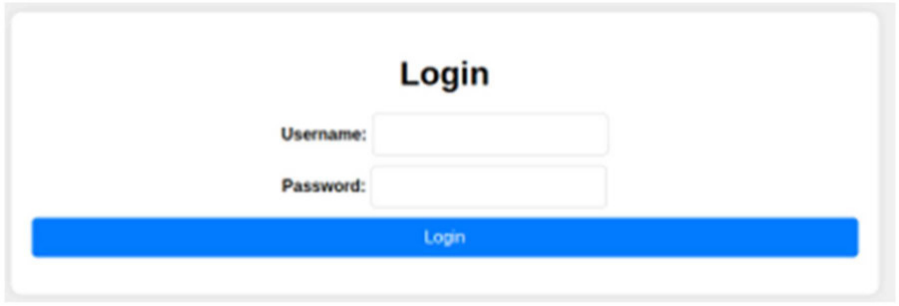
Website login page.

In this study, MediaPipe and Phaser.js libraries provide the right level of abstraction for the work, thus making them an ideal choice. The Phaser.js is a web UI game deployment making it available for players to download, install, or play native to the JavaScript framework. The authentication web UI and home pages were designed using HTML, CSS, and Javascript. The web UI brings together the different modules of the project and makes them work together as a coherent system, as shown in Figure 1. The main reason for implementing a Login system for the website is to provide a personalised score-based report for the user achieved through the login system. The authentication page is shown in Figure 2.

**Figure 3:**
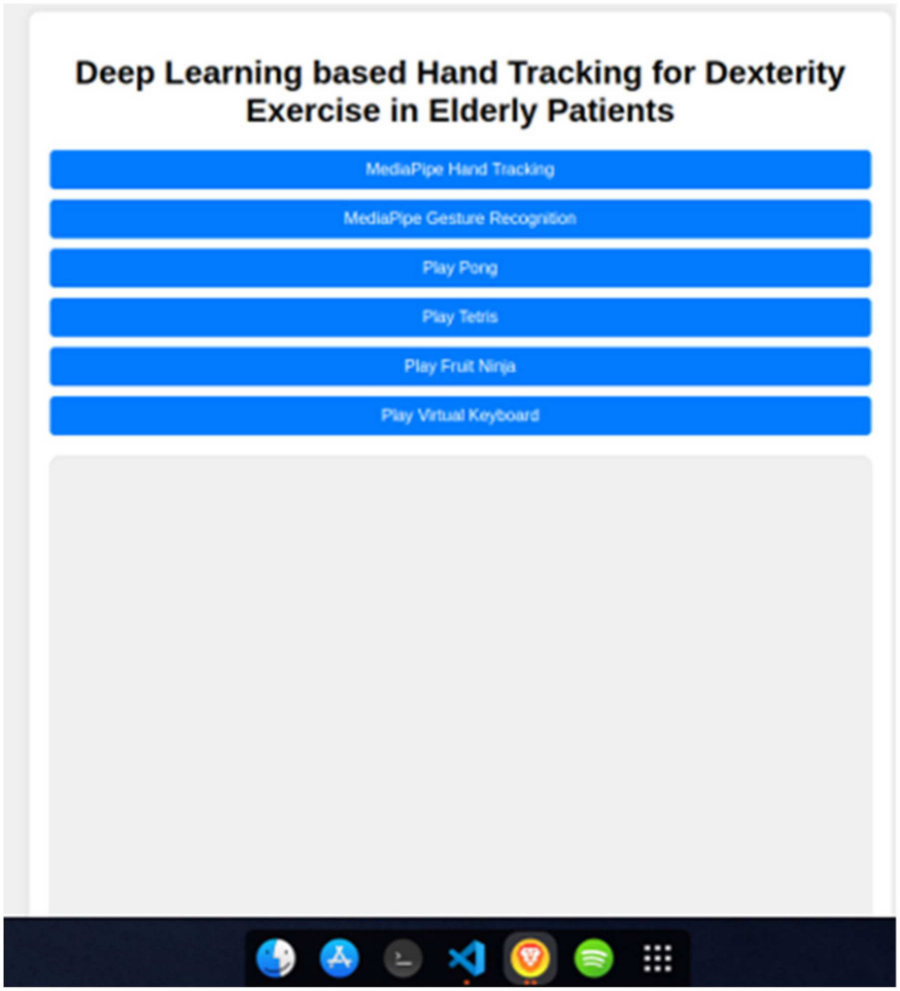
Website home page.

**Figure 4:**
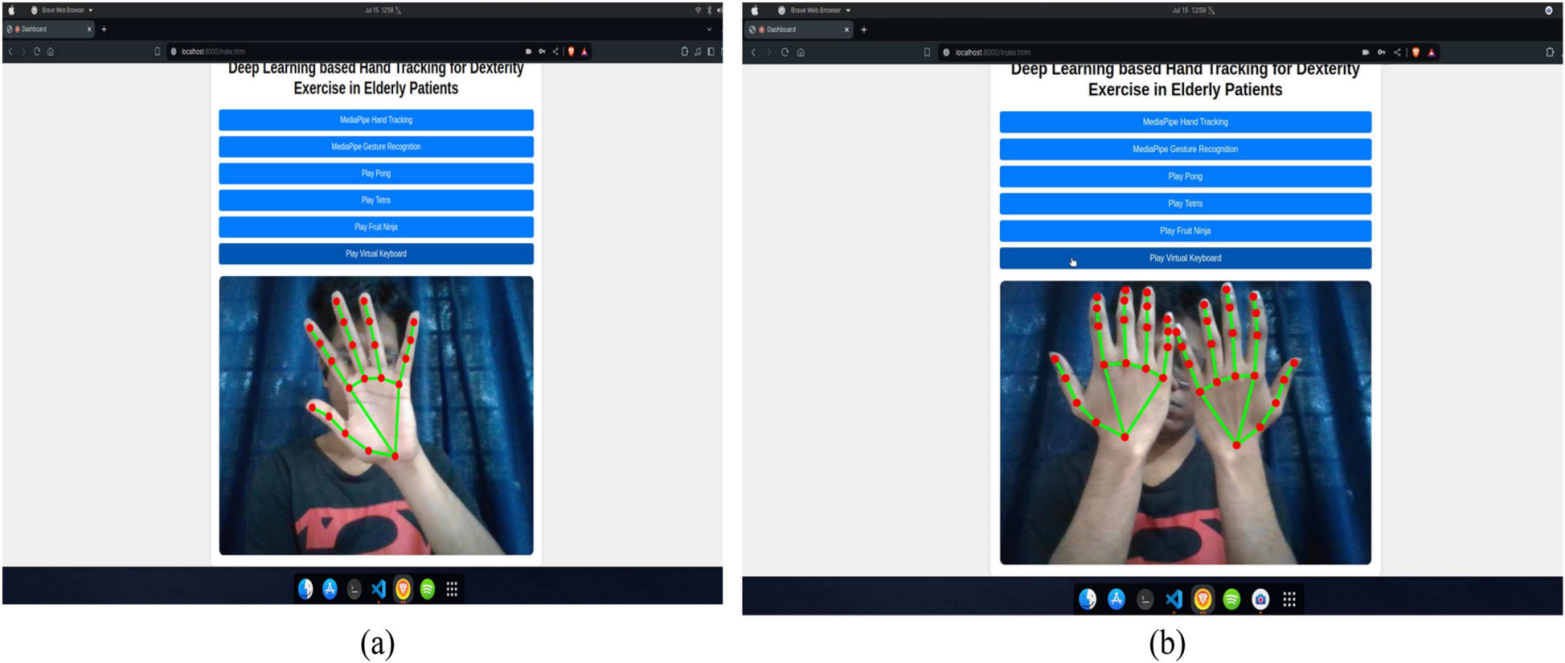
Hand recognition module: **(a)** single hand tracking **(b)** multiple hand tracking.

**Figure 5:**
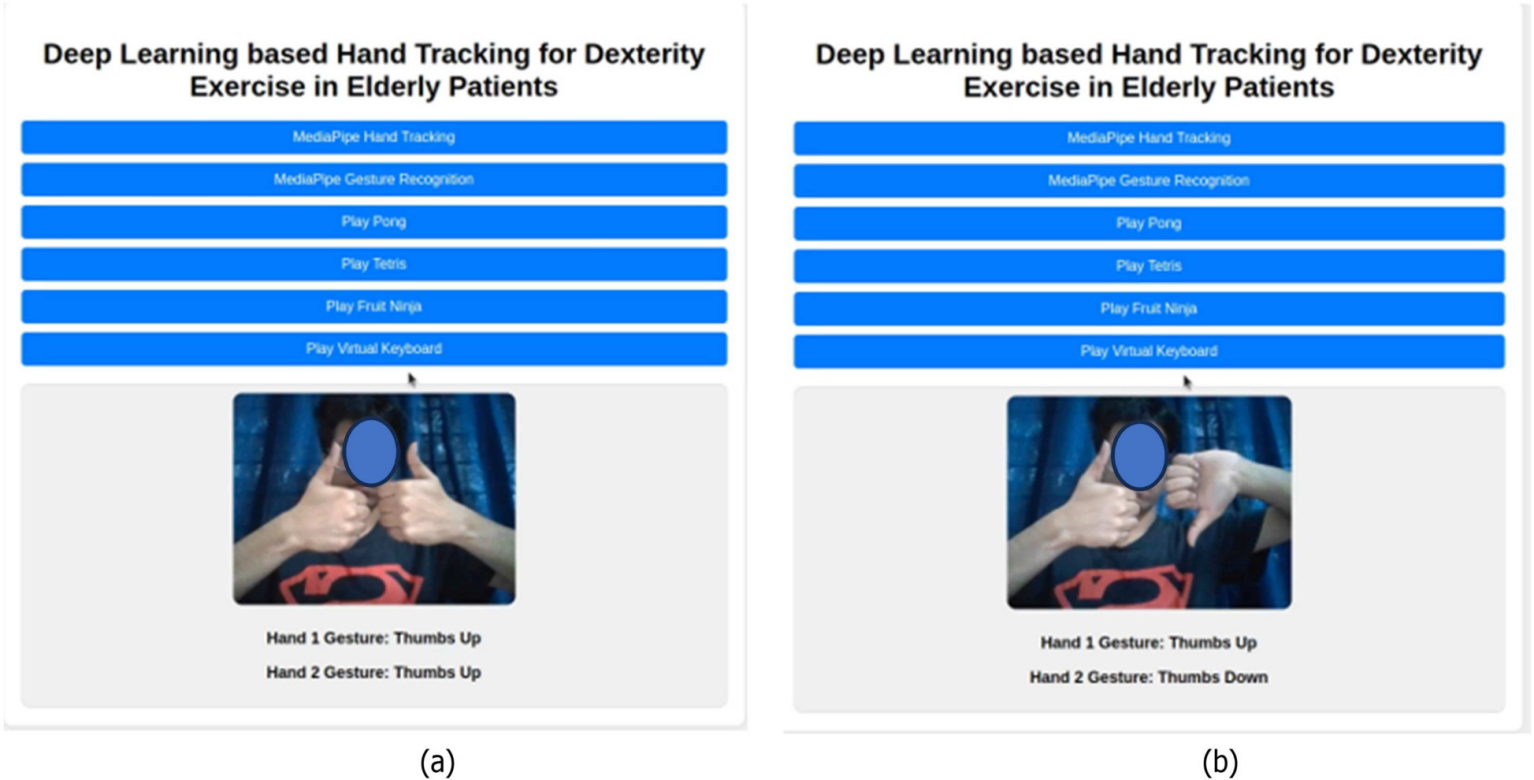
Hand gesture module: **(a)** showing both the hand thumbs up **(b)** showing hand 1 as a thumbs up and hand 2 as a thumbs down.

Upon successful authentication, the user is greeted with the page shown in Figure 3. The following Figures 4a,b demonstrate the use of the hand recognition module, which forms the basis for the gesture recognition system and the control for the games. Figure 5 shows the gesture recognition module in action. These figures demonstrate that the gesture is recognized and displayed on the screen for each hand, whether both hands perform the same gesture or different gestures.

### 4.1 Games development environment

This section discusses the various games developed, the gestures associated with each game, and the functions they should perform. Each game was chosen and designed based on specific therapeutic objectives related to hand function recovery. This system can track various movements, including gross motor skills (like arm sweeps), fine motor skills (such as isolated finger tapping), and cognitive skills (like decision-making and timing).

Four old games have been modified and developed for hand rehabilitation: Pong, Tetris, Fruit Ninja, and Virtual Keyboard.

#### 4.1.1 Pong game

**Figure 6:**
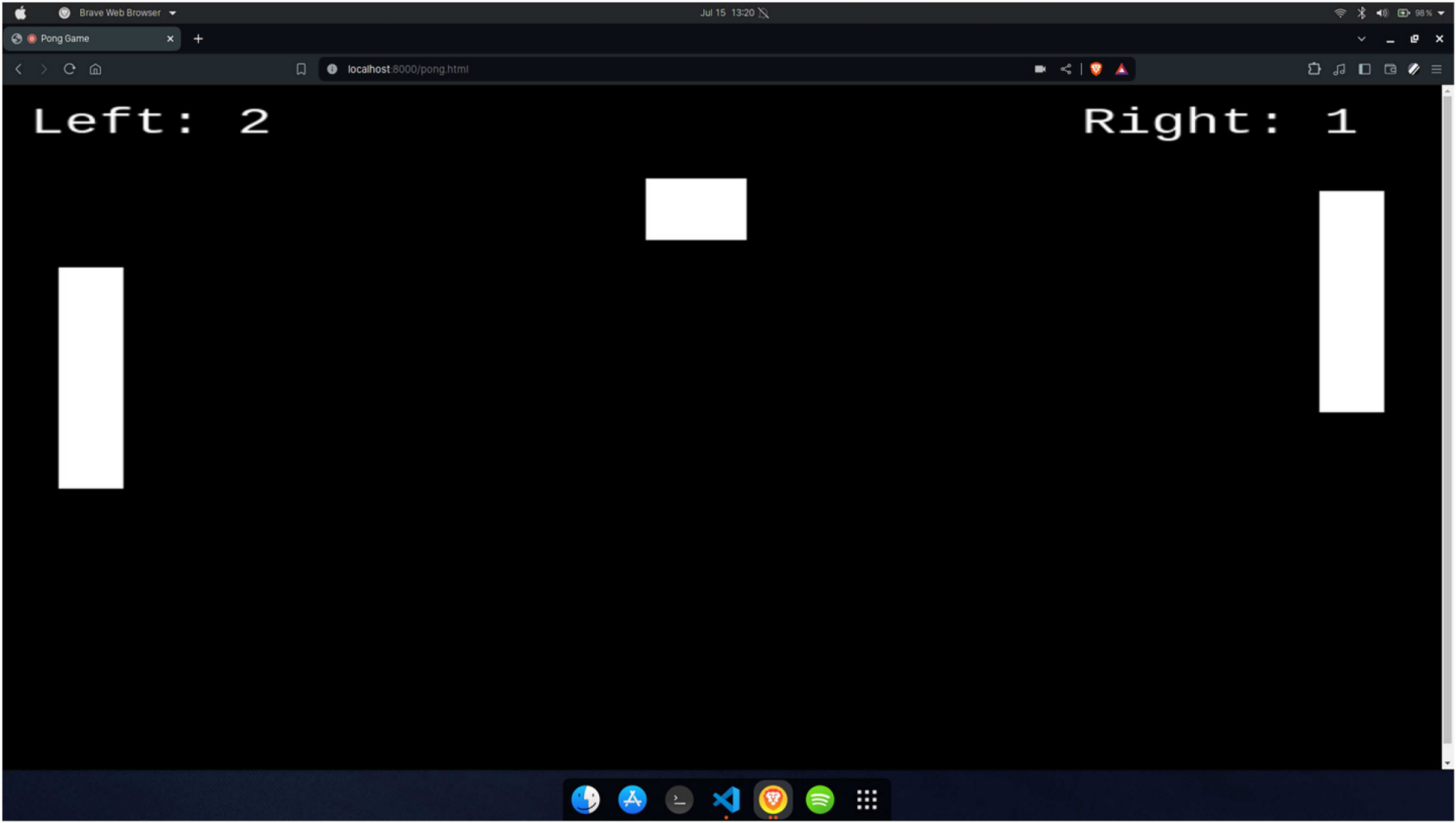
Pong game environment.

The Pong game, shown in Figure 6, can be selected from the home page after user authentication. It is a simplified recreation of the classic Atari game, where two paddles on either side of the screen move vertically to deflect a bouncing ball. A point is scored when one player fails to intercept the ball, causing it to pass through their side, after which the game resets. The gesture recognition module tracks both hands, labelled as Hand 1 and Hand 2 (Figure 5). A thumbs-up gesture moves the paddle upward, while a thumbs-down gesture moves it downward. The game is designed to improve hand-eye coordination and reaction time. As paddle control is not hand-specific, either hand can be used to control either side. With each level, the ball speed gradually increases, requiring quicker gesture responses and anticipation of the ball's movement, intentionally creating a cognitive and reflex-based challenge.

#### 4.1.2 Tetris game

**Figure 7:**
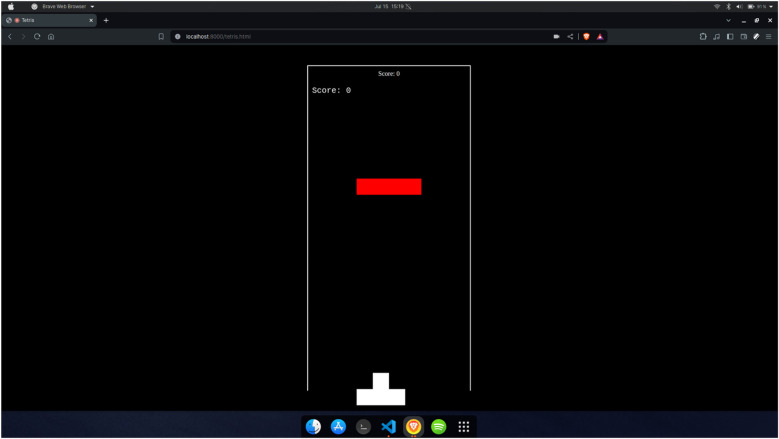
Tetris game environment.

The Tetris game, shown in Figure 7, can be selected from the home page after user authentication. It is a recreation of the classic Atari game, where randomly shaped blocks fall from the top of the screen, and the player must align them to form complete, gap-free rows at the bottom. The gesture recognition module tracks both hands, labelled Hand 1 and Hand 2 (Figure 5), to control the game. For Hand 1, a thumbs-up gesture moves the falling piece to the right, while a thumb-down moves it to the left. For Hand 2, a thumb-up rotates the piece to the right, and a thumb-down rotates it to the left. The game challenges users to make quick, accurate hand movements, requiring fast decision-making to correctly position and orient each block. Once the desired gesture is performed, the user must move their hands away from the screen to prevent unintended changes.

#### 4.1.3 Fruit ninja

**Figure 8:**
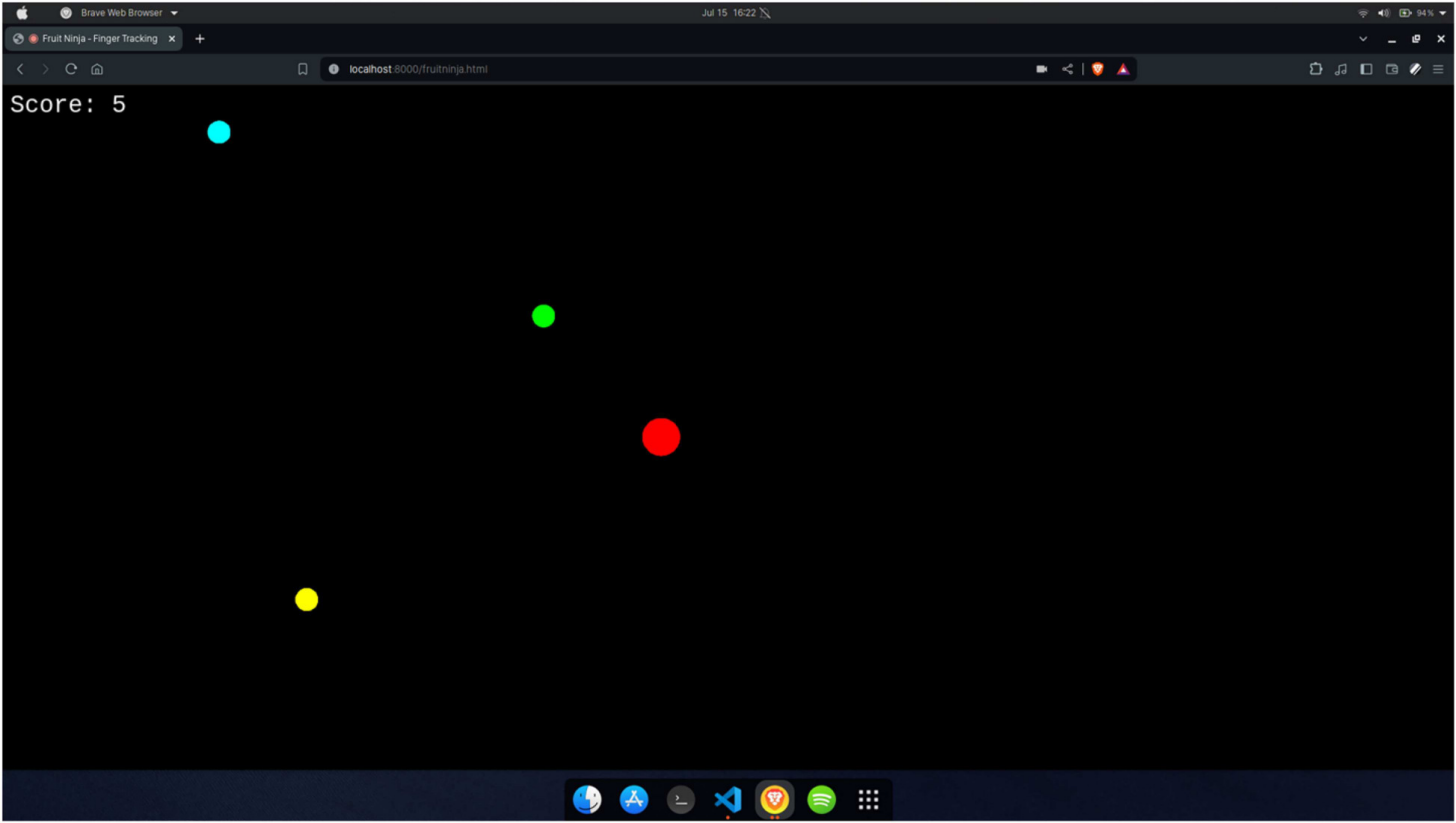
Fruit ninja game.

The Fruit Ninja game, illustrated in Figure 8, can be selected from the home page after user authentication. It is a simplified version of the original Fruit Ninja game, adapted for gesture-based interaction. In this version, randomly colored dots rise from the bottom to the top of the screen, and the user controls a red dot representing a virtual blade using the tip of their index finger. Only one hand is tracked during gameplay. When the red dot overlaps with any of the rising dots, that dot is removed from the screen, and the user scores a point. As the game progresses, the speed of the moving dots gradually increases, requiring the user to maintain focus and react quickly. This game is designed to enhance hand-eye coordination and improve reflexes through engaging and dynamic interaction.

#### 4.1.4 Virtual keyboard game

**Figure 9:**
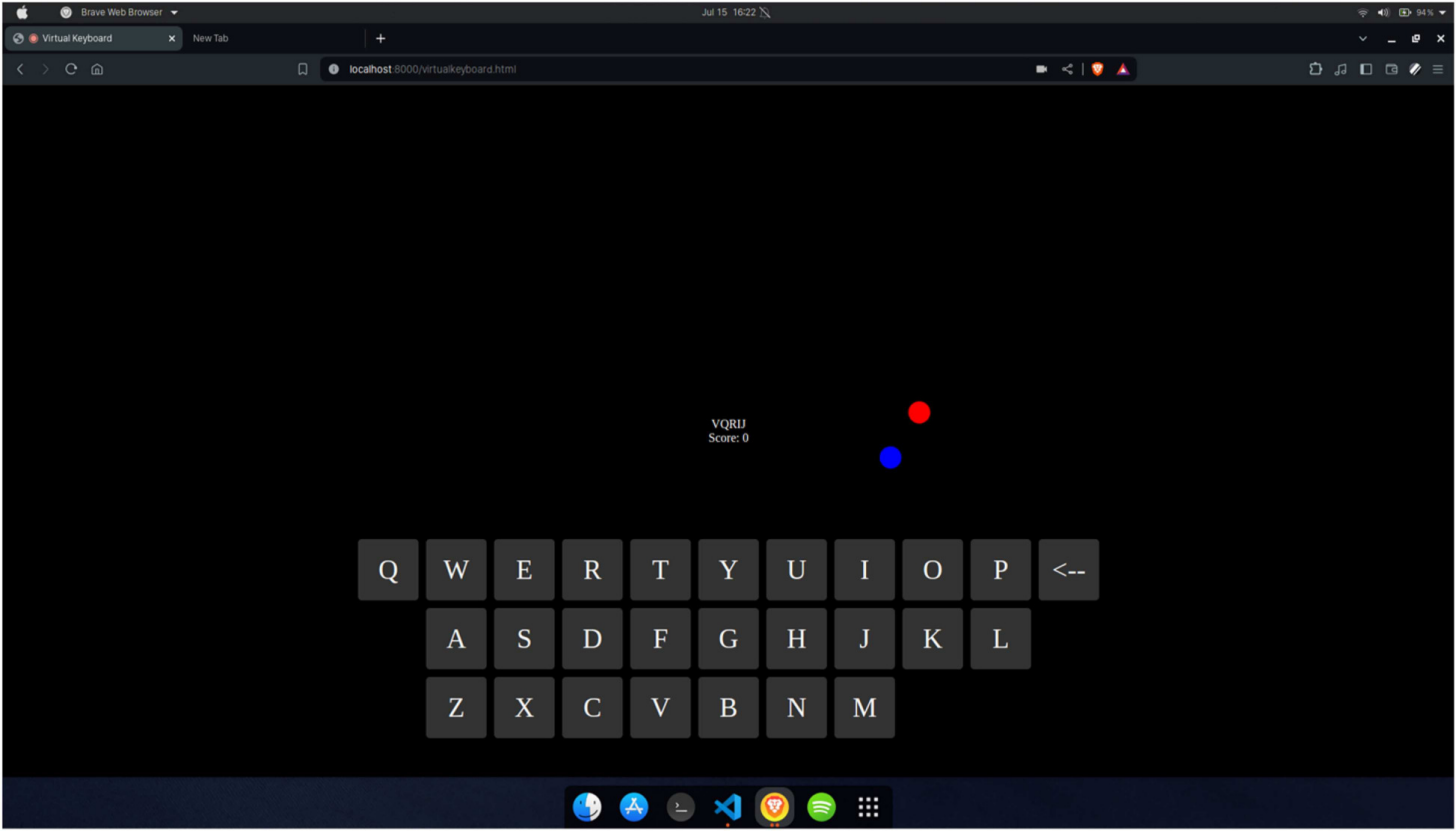
Virtual keyboard game.

The Virtual Keyboard game, as shown in Figure 9, is one of the games that can be selected as one of the games to be played on the home page after the authentication. This game is meant to exercise hand-eye coordination and mental agility. For this game, only one hand is tracked. The red and blue dots (Figure 9) are meant to denote the location of the index finger and the thumb, respectively. Every six seconds, a new random 5-letter word is generated, and the player must type the word at that time before the next word appears. The typing is accomplished by bringing the index finger and the thumb together like a pinch on top of the desired letter. Therefore, the player must maintain hand-eye coordination and be fast due to the time constraints.

## 5 Results and discussion

The experimental dataset was collected from fifteen healthy volunteers (aged 18–60 years) after obtaining informed consent, with approval from the institutional ethics committee. Each participant engaged with all four gesture-controlled rehabilitation games (Pong, Tetris, Fruit Ninja, and Virtual Keyboard). For each game, gesture recognition outputs, accuracy scores, and gameplay performance metrics were recorded. In total, the dataset comprised approximately 3,500 gesture instances across participants, with recognition accuracy ranging between 85%–95%. The dataset was created exclusively for proof-of-concept evaluation and is not intended as a large-scale training corpus. Each participant's performance metrics were recorded as shown in Table 1. Gesture accuracy for each participant per game is calculated using Equation 1,

$$\mathrm{Accuracy}\;(\text{\%})=\frac{C}{G}\times 100$$ (1)

*G* = Total number of gestures performed by the participant in a game.
*C* = Number of gestures correctly recognized for participant.

**Table 1:** Gesture accuracy of four games among individual participants.

Participant ID	Pong accuracy (%)	Tetris accuracy (%)	Fruit ninja accuracy (%)	Virtual keyboard accuracy (%)	SUS score
P01	91.2	86.5	89.7	83	72
P02	93.4	88.1	90.2	84.5	74
P03	88.9	87.3	92	86.2	86
P04	90.5	85.6	88.8	82.4	65
P05	92	89.9	91.3	88.5	93
P06	89.7	87.5	90.1	85.3	84
P07	94.1	90.4	92.8	87.7	70
P08	90.8	86.2	89	84.1	77
P09	87.6	85	88.4	82.9	57
P10	93	91.5	93.1	90.2	88
P11	88.3	84.7	87.5	81.6	93
P12	91.5	88.2	90.9	86.4	87
P13	89.1	86.8	89.3	85	74
P14	92.3	90	91.7	88.1	80
P15	90.6	87.9	90	85.7	79

Across participants, gesture recognition accuracy ranged from 85%–95%.

### 5.1 Statistical comparison of game recognition accuracies

Descriptive results showed mean accuracy of 90.9% (Pong), 87.7% (Tetris), 90.3% (Fruit Ninja), and 85.4% (Virtual Keyboard). While performance was robust overall (all recognition rates were above 85%), analysis revealed a clear influence on task complexity. Games requiring broader, more distinct gestures, such as Pong and Fruit Ninja, showed the highest and most consistent performance, with mean accuracies of 90.9% and 90.3%, respectively. In contrast, the Virtual Keyboard game resulted in the lowest accuracy (85.4%). This suggests that its smaller, more numerous gestures and the demand for finer spatial discrimination posed a greater challenge for the recognition system. The findings support the hypothesis that higher task complexity can impact recognition accuracy, highlighting a key area for future system refinement.

### 5.2 System usability score

The usability of hand-tracking rehabilitation games (Pong, Tetris, Fruit Ninja, and Virtual Keyboard) was assessed with the System Usability Scale (SUS) completed by fifteen participants. SUS scores were calculated using the standard method described by Brooke (1986), yielding a mean score of 78.7 (SD = 10.3). As this exceeds the benchmark value of 68 (23), the system demonstrates good usability. A one-sample *t*-test confirmed that the observed mean was significantly higher than the benchmark, *t* (14) = 4.02, *p* < .01, supporting feasibility of the system as a rehabilitation platform. A summary of the gesture recognition game module is provided in Table 2.

**Table 2:** Summary of gesture recognition game module.

Type of game	Pong	Tetris	Fruit ninja	Virtual keyboard
Purpose	Lateral Hand Movement and Reflex Training	Finger Dexterity and Planning	Reaction Time and Arm Mobility	Precision and Fine Control
Gesture Used	Open palm swipe left/right	Thumb-index pinch to rotate blocks; swipe left/right to move.	Fast swiping gestures in any direction	Pointing with the index finger and fingertip taps
Game Logic	The player's hand controls a paddle. A ball bounces across the screen, and the player must block it.	Standard Tetris rules apply. Blocks must be arranged to complete lines.	Slices falling fruits before they hit the bottom.	A virtual keyboard responds to pointing gestures to type letters.
Gesture Mapping	The horizontal position of the index finger determines paddle location.	Finger spacing thresholds are measured to trigger “rotate” or “drop” actions.	Direction and velocity of swipes are detected from frame-to-frame landmark changes.	When the index fingertip is held steady over a key for >0.5 s, it registers as a key press.
Rehabilitation Aspect: activities like eating and reaching	Encourages lateral shoulder movement, elbow extension, and hand tracking, which are crucial for daily	Involves finer pinch control and timing, supporting skills needed for dressing, buttoning, or writing	Promotes rapid, purposeful arm movements, helping users regain momentum and precision.	Supports skills necessary for typing, touchscreen use, and writing.
Therapeutic Goal	Improve horizontal hand control and visual reflexes	Enhance finger coordination and decision-making.	Improve range of motion and wrist mobility.	Train precision pointing and typing-like skills.

### 5.3 Implications of the study

The study provides implications for physicians and therapists. First, the game console provides a low-cost, non-invasive, camera-only rehabilitation system without the need for wearables or VR headsets, which is ideal for at-home programs. Quantitative tracking of hand activity enables progress monitoring over time. Also, game logs and heatmaps can help tailor rehabilitation intensity and focus areas per patient. Offers remote monitoring and personalized difficulty adjustment. It also helps the patients as it encourages active participation by transforming repetitive tasks into enjoyable challenges. Enables remote rehabilitation, especially for patients in rural or underserved areas. Reduces therapy dropout rates through motivation-driven progress mechanics.

### 5.4 Comparative advantage over low-cost rehabilitation tools

One of the foremost advantages of our camera-based system is its non-invasive and highly accessible nature. In contrast to glove-based systems or robotic exoskeletons, which often entail substantial expense, precise fitting, and challenges in sterilization or portability, whereas our solution capitalizes on a ubiquitous webcam to enable scalable telerehabilitation. While wearable technologies may yield higher raw kinematic fidelity, our deep-learning model attains an accuracy of roughly 85% to 90%, which suffices for the principal goal of facilitating, motivating, and guiding motor exercises in a home environment. These findings suggest that our platform holds promise as a cost-effective adjunct to conventional physiotherapy, particularly in scenarios requiring self-directed or remotely supervised rehabilitation.

In future work, we intend to perform a controlled comparative study with joystick and touchscreen-based rehabilitation systems to more rigorously therapeutic efficacy. These experiments will be conducted with larger and more demographically diverse participant chores. As indicated in Table 3, our approach utilizing a monocular camera and real-time gesture recognition via deep learning offers several distinct advantages relative to prevalent low-cost or commercial rehabilitation technologies, such as wearable sensor systems (e.g., smart gloves or IMU-based devices) and vision systems employing depth cameras (e.g., Kinect).

**Table 3:** Comparative analysis of the proposed monocular camera system vs. alternative low-cost hand rehabilitation tools.

Feature	Monocular camera system (Present study)	Wearable sensor/Glove systems	Other vision systems (e.g., Kinect)
Cost & Accessibility	**Extremely Low:** Requires only a standard webcam or smartphone camera.	**Moderate to High:** Cost associated with sensors, wires, and materials.	**Moderate:** Requires specialized hardware (e.g., depth sensor).
User Experience (Comfort)	**Non-Intrusive:** No physical contact with the patient's hand is required.	**Intrusive:** May restrict hand movement, cause discomfort, or be non-durable.	**Non-Intrusive:** Generally good, but some older systems may require restricted head movement.
Setup & Maintenance	**Minimal:** Simple camera setup.	**Complex:** Requires proper fit, calibration, and cleaning/maintenance of sensors.	**Moderate:** Can be sensitive to background clutter and lighting variations.
Clinical Relevance	Measures movement without physical obstruction, allowing a more **naturalistic assessment** of range of motion.	Captures highly precise kinematic data, but only for the movements the device is designed to measure.	Requires specific environmental controls for optimal performance.

## 6 Conclusion

This research mainly involved developing the gaming console without the need for complex multi-sensor setups. It aims to boost cognitive functions and hand-eye coordination through engaging, game-like exercises that accurately track hand gestures. Implementing games and integrating the controls for these games with the gesture recognition module seems to prove that monocam-based computer vision techniques can be used for dexterity exercises. The design emphasizes simplicity and user-friendliness to ensure easy interaction. Additionally, this research implements a web-based user interface to enhance accessibility as a proof-of-concept feasibility study. The current effort can be further extended to test its efficacy by conducting trials in a randomized control trial in a clinical setting, incorporating adaptive difficulty levels and personalized therapy plans, integrating multimodal sensors (e.g., wearable devices) to complement camera-based tracking, and expanding the platform with additional rehabilitation-focused games designed in collaboration with clinicians. This would lead to identifying potential drawbacks in the current approach and can modify the developed game based on clinician recommendation. SUS provides evidence that your deep learning–based hand-tracking solution meets these real-world constraints, not just laboratory accuracy. The t-test confirmed that the developed rehabilitation platform achieved above-average usability, supporting its feasibility as a patient-friendly system. This statistical validation demonstrates that the system is not only technically viable but also meets the user-experience standards necessary for rehabilitation contexts.

## Data Availability

The raw data supporting the conclusions of this article will be made available by the authors, without undue reservation.

## References

[1] Alarcón-Aldana AC Callejas-Cuervo M Bo APL. Upper limb physical rehabilitation using serious videogames and motion capture systems: a systematic review. Sensors (Basel). (2020) 20(21):5989. 10.3390/s20215989 33105845 PMC7660052

[2] Sandler M Howard A Zhu M Zhmoginov A Chen LC. MobileNetV2: Inverted Residuals and Linear Bottlenecks. *arXiv* [preprint]. *arXiv:1801.0438* (2018). Available online at: https://arxiv.org/abs/1801.04381 (Accessed July 17, 2025).

[3] Dosovitskiy A Beyer L Kolesnikov A Weissenborn D Zhai X Unterthiner T An Image is Worth 16×16 Words: Transformers for Image Recognition at Scale. *arXiv* [preprint]. *arXiv:2010.11929* (2020). Available online at: https://arxiv.org/abs/2010.11929 (Accessed July 17, 2025).

[4] Sandler M Howard A Zhu M Zhmoginov A Chen L-C. MobileNetV2: inverted residuals and linear bottlenecks. In: Forsyth D Laptev I Oliva A Ramanan D Brown MS Morse B, editors. 2018 IEEE/CVF Conference on Computer Vision and Pattern Recognition (CVPR); 2018 Jun 18–23; Salt Lake City, UT, USA. Salt Lake City, UT: IEEE (2018). p. 4510–20. 10.1109/CVPR.2018.00474

[5] He K Zhang X Ren S Sun J. Deep residual learning for image recognition. Proceedings of the IEEE Conference on Computer Vision and Pattern Recognition (2016). p. 770–8. 10.48550/arXiv.1512.03385

[6] Zhu H Chen B Yang C. Understanding why ViT trains badly on small datasets: an intuitive perspective. arXiv [Preprint]. *arXiv:2302.03751* (2023). 10.48550/arXiv.2302.0375

[7] Zhu H Chen B Yang C. Understanding Why ViT Trains Badly on Small Datasets: An Intuitive Perspective. *arXiv* [preprint]. *arXiv:2302.03751* (2023). Available online at: https://arxiv.org/abs/2302.03751 (Accessed July 17, 2025).

[8] Bazarevsky V Grishchenko I Raveendran K Zhu T Zhang F Grundmann M BlazePose: On-device Real-time Body Pose tracking. *arXiv* [preprint]. *arXiv:2006.10204* (2020). Available online at: https://arxiv.org/abs/2006.10204 (Accessed July 17, 2025).

[9] Google. MediaPipe. Available online at: https://github.com/google/mediapipe (Accessed July 17, 2025).

[10] Google. MediaPipe Developers. Available online at: https://developers.google.com/mediapipe (Accessed July 17, 2025).

[11] Alfieri FM da Silva Dias C de Oliveira NC Battistella LR. Gamification in musculoskeletal rehabilitation. Curr Rev Musculoskelet Med. (2022) 15(6):629–36. 10.1007/s12178-022-09797-w 36301514 PMC9789284

[12] Oña ED Pernalete N Jardón A. Towards assessment and rehabilitation of eye-hand coordination using serious games and robotic devices. 2023 IEEE 11th International Conference on Serious Games and Applications for Health (SeGAH); Athens, Greece (2023). p. 1–6. 10.1109/SeGAH57547.2023.10253759

[13] Panaite A Rîsteiu M-N Olar M Leba M Ionica A. Hand rehabilitation—a gaming experience. IOP Conf Ser: Mater Sci Eng. (2019) 572:012092. 10.1088/1757-899X/572/1/012092

[14] Wang HS Hsu C Chiu D Tsai S-N. Using augmented reality gaming system to enhance hand rehabilitation. 2nd International Conference on Education Technology and Computer; Shanghai, China (2010). p. V3-243–46

[15] Jha CK Shukla Y Mukherjee R Rathva P Joshi M Jain D. A glove-based virtual hand rehabilitation system for patients with post-traumatic hand injuries. IEEE Trans Biomed Eng. (2024) 71(7):2033–41. 10.1109/TBME.2024.3360888 38294922

[16] Bouatrous A Zenati N Meziane A Hamitouche C. A virtual reality game for hand rehabilitation after stroke. CEUR Workshop Proceedings. Vol. 3616. Available online at: https://ceur-ws.org/Vol-3616/paper3.pdf

[17] Bostanci H Emir A Tarakci D Tarakci E. Video game-based therapy for the non-dominant hand improves manual skills and grip strength. Hand Surg Rehabil. (2020) 39(4):265–9. 10.1016/j.hansur.2020.02.011 32247654

[18] Alimanova M Borambayeva S Kozhamzharova D Kurmangaiyeva N Ospanova D Tyulepberdinova G Gamification of hand rehabilitation process using virtual reality tools: using leap motion for hand rehabilitation. 2017 First IEEE International Conference on Robotic Computing (IRC); Taichung, Taiwan. (2017). p. 336–9

[19] Chiu HC Ada L Lee HM. Upper limb training using wii sports resort™ for children with hemiplegic cerebral palsy: a randomized, single-blind trial. Clin Rehabil. (2014) 28(10):1015–24. 10.1177/0269215514533709 24849793

[20] Lansberg MG Legault C MacLellan A Parikh A Muccini J Mlynash M Home-based virtual reality therapy for hand recovery after stroke. PM R. (2022) 14(3):320–8. 10.1002/pmrj.12598 33773059

[21] Luna-Oliva L Ortíz-Gutiérrez R Cano de la Cuerda R Martínez-Piédrola R Alguacil I Sánchez-Camarero C Kinect Xbox 360 as a therapeutic modality for children with cerebral palsy in a school environment: a preliminary study. NeuroRehabilitation. (2013) 33(4):513–21. 10.3233/NRE-131001 24018364

[22] Brooke J. SUS: a “quick and dirty” usability scale. In: Jordan PW Thomas B Weerdmeester BA McClelland AL, editors. Usability Evaluation in Industry. London: Taylor & Francis (1996). p. 189–94.

[23] Bangor A Kortum PT Miller JT. An empirical evaluation of the system usability scale. Int J Hum Comput Interact. (2008) 24(6):574–94. 10.1080/10447310802205776

