# Supplementary material for: Beyond the joystick: deep learning games for hand movement recovery
Source: Front Rehabil Sci. 2025 Nov 12;6:1653302. doi: 10.3389/fresc.2025.1653302 (PMC12647053; doi:10.3389/fresc.2025.1653302)
Supplement: Supplementary file 1 [file Table1.docx]

Algorithm 1: Pseudocode for the pong game

| 1. Initialize game:  - Set game configuration (type, width, height, background color, parent, scene)  - Initialize variables: board, currentPiece, nextPiece, leftHandGesture, rightHandGesture, score  2. Preload assets:  - Load necessary game assets (if any)  3. Create game scene:  - Initialize board to 20x10 grid with all cells set to 0  - Call spawnPiece()  - Start game tick timer (500ms interval, calls gameTick)  - Start gesture recognition timer (1000ms interval, calls runGestureRecognition)  - Set end game timer (60 seconds, calls endGame)  - Initialize score text  - Initialize graphics for rendering  4. gameTick():  - If leftHandGesture is "up":  - Call rotatePiece()  - If rightHandGesture is "up":  - Call movePiece(-1, 0) // Move left  - If rightHandGesture is "down":  - Call movePiece(1, 0) // Move right  - Else:  - Call movePiece(0, 1) // Move down  - Reset hand gestures (leftHandGesture, rightHandGesture)  - Call renderBoard()  5. movePiece(dx, dy):  - If no collision at new position (currentPiece.x + dx, currentPiece.y + dy):  - Update currentPiece position (x += dx, y += dy)  - Else if dy != 0 (moving down):  - Call lockPiece()  - Call clearLines()  - Call spawnPiece()  - If collision at new position (currentPiece.x, currentPiece.y):  - Call endGame()  6. rotatePiece():  - Rotate currentPiece shape  - If no collision at new position:  - Update currentPiece shape  7. checkCollision(shape, offsetX, offsetY):  - For each cell in shape:  - If cell is occupied and board cell at (offsetX, offsetY) is also occupied:  - Return true  - Return false  8. lockPiece():  - For each cell in currentPiece shape:  - If cell is occupied:  - Set corresponding board cell to occupied  9. clearLines():  - For each row in board:  - If row is full:  - Remove row  - Add new empty row at top  - Update score  10. renderBoard():  - Clear previous graphics  - For each cell in board:  - If cell is occupied:  - Render cell  - For each cell in currentPiece shape:  - If cell is occupied:  - Render cell  11. runGestureRecognition():  - Placeholder for gesture recognition logic  - Update leftHandGesture and rightHandGesture based on detected gestures  12. endGame():  - Pause game  - Display final score |
| --- |

Algorithm 2: Pseudocode for the Tetris game

| 1. Initialize game:  - Set game configuration (type, width, height, background color, parent, scene)  - Initialize variables: board, currentPiece, nextPiece, leftHandGesture, rightHandGesture, score  2. Preload assets:  - Load necessary game assets (if any)  3. Create game scene:  - Initialize board to 20x10 grid with all cells set to 0  - Call spawnPiece()  - Start game tick timer (500ms interval, calls gameTick)  - Start gesture recognition timer (1000ms interval, calls runGestureRecognition)  - Set end game timer (60 seconds, calls endGame)  - Initialize score text  - Initialize graphics for rendering  4. gameTick():  - If leftHandGesture is "up":  - Call rotatePiece()  - If rightHandGesture is "up":  - Call movePiece(-1, 0) // Move left  - If rightHandGesture is "down":  - Call movePiece(1, 0) // Move right  - Else:  - Call movePiece(0, 1) // Move down  - Reset hand gestures (leftHandGesture, rightHandGesture)  - Call renderBoard()  5. movePiece(dx, dy):  - If no collision at new position (currentPiece.x + dx, currentPiece.y + dy):  - Update currentPiece position (x += dx, y += dy)  - Else if dy != 0 (moving down):  - Call lockPiece()  - Call clearLines()  - Call spawnPiece()  - If collision at new position (currentPiece.x, currentPiece.y):  - Call endGame()  6. rotatePiece():  - Rotate currentPiece shape  - If no collision at new position:  - Update currentPiece shape  7. checkCollision(shape, offsetX, offsetY):  - For each cell in shape:  - If cell is occupied and board cell at (offsetX, offsetY) is also occupied:  - Return true  - Return false  8. lockPiece():  - For each cell in currentPiece shape:  - If cell is occupied:  - Set corresponding board cell to occupied  9. clearLines():  - For each row in board:  - If row is full:  - Remove row  - Add new empty row at top  - Update score  10. renderBoard():  - Clear previous graphics  - For each cell in board:  - If cell is occupied:  - Render cell  - For each cell in currentPiece shape:  - If cell is occupied:  - Render cell  11. runGestureRecognition():  - Placeholder for gesture recognition logic  - Update leftHandGesture and rightHandGesture based on detected gestures  12. endGame():  - Pause game  - Display final score |
| --- |

Algorithm 3: Pseudocode for the Fruit Ninja game

| 1. Initialize game configuration:  - Set type to Phaser.AUTO  - Set width to window.innerWidth  - Set height to window.innerHeight  - Set parent to 'game-container'  - Set backgroundColor to '#000000'  - Define scene with methods: preload, create, update  2. Create Phaser game instance:  - Initialize game with config  3. Initialize variables:  - Set indexFingerTip to { x: 0, y: 0 }  - Initialize empty balls array  - Set score to 0  - Initialize scoreText  4. preload():  - No assets to preload  5. create():  - Initialize video element  - Initialize MediaPipe Hands with options  - Set MediaPipe Hands result callback  - Initialize camera for video feed and start capturing  - Initialize graphics for rendering  - Initialize score text display  - Set timer to call createBall() at regular intervals (every 1000ms)  6. update():  - Clear previous graphics  - Draw a large red circle at indexFingerTip position  - Update each ball's position:  - Move ball upwards by its speed  - If ball goes off-screen, remove it from balls array  - Draw ball at new position  - Check for collisions:  - For each ball:  - Calculate distance between indexFingerTip and ball  - If distance < threshold (collision detected):  - Increase score  - Update score text  - Remove ball from balls array  7. createBall():  - Generate random color for ball  - Set random initial x position at bottom of screen  - Set y position to window.innerHeight  - Set random speed for ball  - Add new ball to balls array  8. onResults(results):  - If hand landmarks detected:  - Get index fingertip position (landmark 8)  - Convert normalized coordinates to screen coordinates  - Update indexFingerTip with new position |
| --- |

Algorithm 4: Pseudocode for the Virtual Keyboard game

| 1. Initialize game configuration:  - Set canvas dimensions and context  - Get display elements and keys  - Initialize pressedKeys, score, randomString, gameInterval, randomStringInterval  2. Initialize MediaPipe Hands:  - Set MediaPipe options for detection and tracking  - Define callback function for hand results  - Initialize camera for video feed and start capturing  3. onResults(results):  - Clear canvas  - If hand landmarks detected:  - Get index finger tip and thumb tip positions  - Convert normalized coordinates to screen coordinates  - Draw circles at fingertip positions  - Check for key press based on fingertip positions  4. drawCircle(x, y, color):  - Draw circle on canvas at (x, y) with given color  5. checkKeyPress(indexTipX, indexTipY, thumbTipX, thumbTipY):  - Calculate distance between index finger tip and thumb tip  - If distance < threshold (click detected):  - For each key:  - Get key's bounding box  - Check if index finger tip is within key bounds  - If key pressed:  - Update pressedKeys  - Update display with pressedKeys  6. generateRandomString(length):  - Initialize empty result string  - For length times:  - Append random character (A-Z) to result string  - Return result string  7. updateRandomString():  - If pressedKeys matches randomString:  - Increment score  - Update score display  - Reset pressedKeys and display  - Generate and display new randomString  8. startGame():  - Generate and display initial randomString  - Set interval to call updateRandomString() periodically  - Set timer to end game after specified duration |
| --- |
